# Erratum to: The effect of chemotherapy on expression of folate receptor-alpha in ovarian cancer

**DOI:** 10.1007/s13402-012-0084-6

**Published:** 2012-06-12

**Authors:** Lucia M. A. Crane, Henriette J. G. Arts, Marleen van Oosten, Philip S. Low, Ate G. J. van der Zee, Gooitzen M. van Dam, Joost Bart

**Affiliations:** 1 Department of Surgery, Division of Surgical Oncology, University of Groningen, University Medical Center Groningen, Groningen, the Netherlands; 2 Department of Obstetrics and Gynaecology, Division of Gynaecologic Oncology, University of Groningen, University Medical Center Groningen, Groningen, the Netherlands; 3 Department of Chemistry, Purdue University, West Lafayette, IN 47907 USA; 4 Department of Pathology and Medical Biology, University of Groningen, University Medical Center Groningen, P.O. Box 30.001, 9700 RB Groningen, the Netherlands

**Erratum to: Cell Oncol. (2011)**

**DOI 10.1007/s13402-011-0052-6**

The affiliations of the authors of the above mentioned article, published in Cellular Oncology, volume 35, issue 1, February 2012, pp. 9–18, should read:

L. M. A. Crane . M. van Oosten . G. M. van Dam Department of Surgery, Division of Surgical Oncology, University of Groningen, University Medical Center Groningen, Groningen, the Netherlands

H. J. G. Arts . A. G. J. van der Zee Department of Obstetrics and Gynaecology, Division of Gynaecologic Oncology, University of Groningen, University Medical Center Groningen, Groningen, the Netherlands

P. S. Low Department of Chemistry, Purdue University, West Lafayette, IN 47907, USA

J. Bart (*Corresponding author) Department of Pathology and Medical Biology, University of Groningen, University Medical Center Groningen, P.O. Box 30.001, 9700 RB Groningen, the Netherlands

